# Psychological impact of COVID‐19 pandemic on healthcare workers in China Xi’an central hospital

**DOI:** 10.1002/brb3.2028

**Published:** 2021-01-06

**Authors:** Nan Wang, Yongqin Li, Qiaoxia Wang, Caihong Lei, Yuanyuan Liu, Shanshan Zhu

**Affiliations:** ^1^ Department of Infectious Diseases Xi’an Central Hospital Xi’an Shaanxi China

**Keywords:** COVID‐19, healthcare workers, mental health, responsibility

## Abstract

**Objective:**

COVID‐19 significantly altered our routine, lifestyle, and stress level across the globe. This study investigated the psychological impact of COVID‐19 on healthcare workers in China Xi'an Center hospital.

**Methods:**

A modified online questionnaire of Psychological Status and the General Health Questionnaire (GHQ‐12) was provided to 1,967 healthcare workers during the COVID‐19 pandemic. Participation was voluntary, and the responses were anonymous. The survey lasted for 2 weeks, and the GHQ‐12 was completed every other day. The data were collected automatically and electronically and then statistically analyzed.

**Results:**

The 431 (21.9%) responders included 214 nurses (49.7%), 146 clinicians (33.9%), 29 pharmacists (6.7%), 15 medical technicians (3.5%), 17 administrative staff (3.9%), and 10 other departments (2.3%). Of these, 46.2% had 10 years of work experiences or more and 78.2% were married. Work experience increased emotional stress as 23% of participants with 10 years or more of experience exhibited higher stress compared to those with fewer than 3 years of work experience (7.5%). Moreover, 33.3% of participants who worked in or were exposed to the affected areas of the pandemic experienced psychological stress. Overall, this study identified four factors that were significantly associated with psychological stress: (a) work experience (OR 2.99; 95% CI: 1.06 to 8.41); (b) change in job position (OR 1.99; 95% CI: 1.10 to 3.59); (c) change in lifestyle (OR 4.06; 95% CI: 1.81 to 9.10); and (d) need for psychological counseling (OR 3.07; 95% CI: 1.62 to 5.82).

**Conclusions:**

The COVID‐19 pandemic has increased psychological stress among healthcare workers with 10 years or more work experiences and who recently experienced a career position change.

## Graphical abstract

For the epidemic of COVID‐19, more attention should be paid to the mental health of healthcare workers with more work experience and changing job positions; in addition to providing interventions for psychological impact, more personal and family support for healthcare workers is necessary.

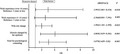

## 1 INTRODUCTION

Coronavirus 2019 (COVID‐19) is an infectious respiratory disease caused by the severe acute respiratory syndrome coronavirus 2 (SARS‐CoV‐2) (Chen et al., 2020). Since late in 2019, there have been more than 54 million cases reported from over 191 countries and territories and more than 1,317,812 deaths globally (Data from Johns Hopkins University). In China alone, there were official reports of 86,346 confirmed cases and 4,634 deaths as of 16 November 2020 (National Health Commission of the People's Republic of China). However, the proportion of confirmed severe cases among the healthcare workers was 17.7% in Wuhan, 10.4% in Hubei, and 7.0% in regions outside Hubei. Zhang et al. of the Chinese Center for Disease Control and Prevention analyzed the epidemiological characteristics of COVID‐19 (Zhang, 2020). This work showed that among the 422 medical institutions providing diagnosis and treatment services for COVID‐19 patients, 3,019 healthcare workers contracted the disease with 1,716 confirmed cases and 5 deaths (a crude mortality rate of 0.3%). On 22 January, our Xi'an Central Hospital was identified as one of the designated hospitals for COVID‐19 patients. Immediately after this designation, our hospital provided healthcare workers with centralized training, organized a specialized expert team to fight COVID‐19, and mobilized healthcare workers to participate in a triage station, fever clinic, and quarantine ward. In our hospital, healthcare workers screen patients with a fever for the possibility of having contracted COVID‐19 and admit the patients with the virus for hospitalization and treatment. To do this, many healthcare workers that are not work in the infectious department also participate in the screening and treatment of COVID‐19 patients, but need to be trained for such a purpose.

The COVID‐19 pandemic has not only significantly affected our community, society, country, and the whole world, but has also been responsible for adverse psychological impacts on our healthcare workers. Our healthcare workers have continued with their duty to care for patients, but are also at risk of becoming infected, due to the combination of the sudden outbreak and the little information known regarding COVID‐19 characteristics, infection level, and severity. The several recent studies (Lai et al., 2020; Mohd Fauzi et al., 2020; Rossi et al., 2020) showed that healthcare workers in hospitals equipped with fever clinics or wards for COVID‐19 patients had experienced psychological burden. Other healthcare workers with different positions and duties also face varying levels of psychological impact. A previous study showed that 50.7%, 44.7%, 36.1%, and 73.4% of epidemiologists and healthcare works experienced depression, anxiety, sleep disorder, and stress, respectively (Liu et al., 2020), while the first‐line healthcare workers experienced even higher incidence rates and higher risk of psychological stress (Que et al., 2020; TsamakisK et al., 2020). The psychological stress negatively impacted their psyche, sleep quality, and work efficiency (Wu et al., 2020). Thus, a better understanding of the psychological impact on healthcare workers and their adjustment to treating COVID‐19 patients could help provide them with appropriate psychological support and lead to better patient care. In this study, we assessed the psychological impact of COVID‐19 on healthcare workers in the China Xi'an Central hospital. This work aims to provide useful information regarding the factors that induce stress on healthcare workers. The results of this study may help provide more appropriate and encompassing information for the psychological support of those healthcare workers experiencing stress and improve the quality of patient care.

## 2 SUBJECTS AND METHODS

### 2.1 Study participants

An anonymous cross‐sectional study was performed using the social media platform‐based (WeChat) survey program, “Questionnaire Star,” between 1 and 14 February 2020. The data collection period was restricted to only these 2 weeks. We included questionnaires from all healthcare workers from Xi'an Central Hospital (Xi'an, China) who held a position involving clinical patient care, medical technologies, or administrative management. First, we sent the informed consent form electronically to all healthcare workers in our hospital, and those who agreed to participate in our study later received our study questionnaire. The exclusion criteria were as follows: (a) participant not a healthcare worker at Xi'an Central Hospital, (b) absentee due to disease or vacation, (c) participant not a user of WeChat, and (d) participant did not complete the assessment.

### 2.2 Questionnaire and data collection

To collect data from the included healthcare workers, we utilized an online electronic questionnaire survey. Before initiating the formal study, we first interviewed 23 healthcare workers from the infectious disease department (including 9 doctors and 14 nurses) to test data acquisition, analyze the potential influencing factors, and finalize our questionnaire. We then revised and finalized the questionnaire by consulting with clinical psychiatrists (Peng Wang and his associates). The final questionnaire included three parts, that is, the first part involved general information of the respondents, such as gender, age, education level, work experience, current position, marital status, and children. The second part contained 24 items, including open and closed questions regarding the type of hospital support most needed by respondents, disease prevention and control measures, and changes in the respondents’ work and lifestyle. The third part contained the 12‐item General Health Questionnaire (GHQ‐12), a psychometric screening tool originally designed by Goldberg et al. to identify common psychiatric conditions (Goldberg, 1972). GHQ‐12 is also frequently used to assess psychological distress in a population (Cuéllar‐Flores et al., 2014; Gómez‐Salgado et al., 2020; Ogundipe et al., 2014), which includes 12 sections of questions each assessed with a four‐point Likert scale, and is considered valid for use on adults and adolescents. According to the World Health Organization (WHO) guidelines, the GHQ‐12 questionnaire is frequently used with the 0–0–1–1 scoring method where the first two and last two choices are scored as 0 and 1 point, respectively, leading to the total score ranging between 0 and 12 points. The General Health Questionnaire developed by Goldberg et al. have been used in various countries and was previously used in studies of other SARS‐like epidemics (Tam et al., 2004). In this study, we used three points as the cut‐off value where three points or more suggested a mental health problem (Bizu et al., 2015; Tomoyuki, 2015; Wang et al., 2012) (the higher the score, the more significant the mental problems are). We distributed these questionnaires electronically to all included healthcare workers (*n* = 1,967) in the hospital and collected 431 (21.9%) responses. This study was approved by the Ethics Committee of Xi'an Central Hospital (Xi'an, China), and all responders provided written informed consent form before participating in this study.

The electronic questionnaires were sent via either mobile phone or computer terminal but not both to prevent repeat filings. During the survey period, the responders provided data every 2 days for 2 weeks. The feedback questionnaires were also reviewed every 2 days and the incomplete questionnaires were eliminated electronically to insure only full datasets were acquired. The data were collected through an online survey platform and the responses to the questionnaires were automatically encoded and organized in the background to avoid errors caused by manual entry.

### 2.3 Statistical analysis

The counting data are summarized as the composition ratio (the GHQ‐12 data are “two class variance” and counted as numbers between 0 and 12), and the difference among groups was analyzed using a chi‐square or Fisher's exact test. The variables exhibiting a significant difference were counted as independent variables, for example, the GHQ‐12 threshold score was counted as a dependent variable and statistically analyzed using binary logistic regression. All statistical analyses were performed using the SPSS 23.0 for Windows software package and the statistical significance level was set at *p ≤ *.05.

## 3 RESULTS

### 3.1 Demographic characteristics and GHQ‐12 scores

In this study, we collected a total of 431 (21.9%) responses, including 94 males (21.8%) and 337 females (78.2%). Moreover, the participants included 214 nurses (49.7%), 146 clinicians (33.9%), 29 pharmacists (6.7%), 15 medical technicians (3.5%), 17 administrative staff (3.9%), and 10 other departments (2.3%). Of these, 46.2% had work experience of 10 years or more, 78.2% were married, and 70.3% had at least one child. These demographic data are shown in Table 1.

**TABLE 1:** Demographic characteristics from all responders (*n* = 431)

Variables	*n*	(%)
Gender		
Male	94	(21.8)
Female	337	(78.2)
Age (years)		
18–24	19	(4.4)
25–30	130	(30.2)
31–40	179	(41.5)
41–50	74	(17.2)
51–60	29	(6.7)
Education level		
Two‐year college and below	115	(26.7)
Bachelor	190	(44.1)
Master and above	126	(29.2)
Work experience (years)		
≤3	67	(15.5)
4–10	165	(38.3)
≥10	199	(46.2)
Marital status		
Married	337	(78.2)
Single	87	(20.2)
Divorced	7	(1.6)
Children		
0	128	(29.7)
1	222	(51.5)
2	81	(18.8)
Specialty		
Administration	17	(3.9)
Nurse	214	(49.7)
Clinician	146	(33.9)
Others	10	(2.3)
Pharmacist	29	(6.7)
Medical imaging technician	15	(3.5)

**FIGURE 1:**
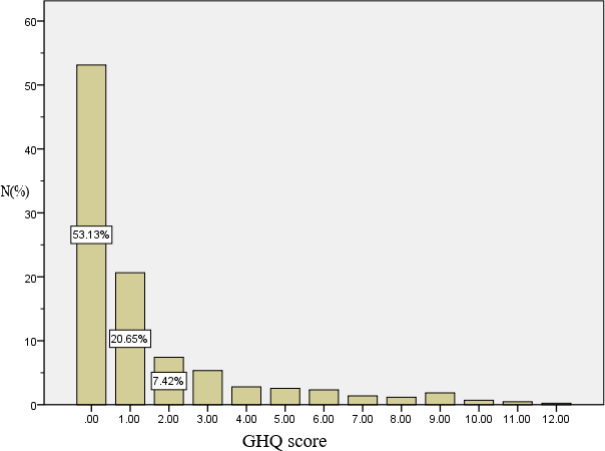
Distribution of total scores of GHQ‐12 among responders (*n* = 431)

**FIGURE 2:**
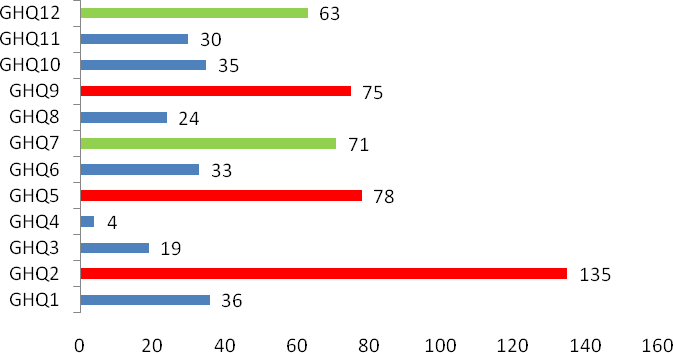
Numbers of responders who scored 1 for each category in the GHQ‐12

Of the 431 responders who completed the GHQ‐12 every other day for the full 2 weeks, 81 (18.8%) scored above the threshold of 3 (Figure 1), indicating the existence of different degrees of emotional distress. The numbers of responders with a score of 1 for each category are summarized in Figure 2. Of note, 135 responders (31.3%) scored 1 for Item 2 “insomnia due to worry,” 78 (18.1%) for Item 5 “always tense,” and 75 (17.4%) for Item 9 “feeling distressed and worried.” These three items fall into the anxiety/depression dimension of the GHQ scale and indicate that anxiety and depression were prominent among healthcare workers for the duration of the 2‐week survey. Moreover, 71 (16.5%) scored a 1 for Item 7 “can't enjoying daily activities” and 63 (14.6%) scored a 1 for Item 12 “feeling that everything isn't going well.” These two items belong to the low social function dimension of the GHQ scale and indicate that the emotional distress affected their social functions.

### 3.2 Comparison of the different GHQ‐12 scores among responders

There was a statistically significant difference (χ^2^ = 8.113, *p* = .017) in work experience for those participants who exhibited a score greater than 3. In this sense, only 7.5% of participants with less than 3 years of work experience scored above the threshold of greater than 3 whereas 23.1% of participants with 10 years or more work experiences scored above than 3. Moreover, 33.3% of those who worked in or were exposed to the affected areas of the epidemic scored 3 or more, whereas only 17.7% of participants with no such an exposure scored a 3 or more (χ^2^ = 4.467; *p* = .035).

Moreover, the method of acquiring epidemic information also affected the GHQ‐12 scores. The proportion of healthcare workers with GHQ‐12 ≤ 3 who obtained COVID‐19 epidemic‐related information through text message, community information, and hospital department training was higher than those who did not receive such information. Furthermore, change in job position also significantly affected the GHQ‐12 scores (χ^2^ = 7.285; *p* = .007) as 27.5% of participants reported a score of 3 or more after a job change, whereas only 15.8% reported a score of 3 or more without job changes. Job position also affected the GHQ‐12 scores. Only 14.3% of responders who worked at the triage station reported a score of 3 or more, whereas 47.4% of responders who worked at the fever clinic reported the same (Fisher = 10.75; *p* = .041).

In addition, emotional distress was also higher among those who believed “the epidemic has changed personal or family lifestyle” (χ^2^ = 9.378; *p* = .002). However, responders who denied “the epidemic to play a positive role in improving the doctor–patient relationship” seemed to be more inclined to experience emotional distress (χ^2^ = 4.462; *p* = .035). The corresponding data are shown in Table 2.

**TABLE 2:** Association of different categories with GHQ‐12 scores

Variables	GHQ−12 scores [*n* (%)]		$\chi^{2}$	*p* value
	<3	≥3		
Working experience				
≤3 years	62 (92.5)	5 (7.5)	8.113	.017
4–10 years	135 (81.8)	30 (18.2)		
≥10 years	153 (76.9)	46 (23.1)		
Exposure to epidemic area or people				
No	330 (82.3)	71 (17.7)	4.467	.035
Yes	20 (66.7)	10 (33.3)		
The channel to get COVID−19 information				
TV				
No	156 (77.6)	45 (22.4)	3.189	.074
Yes	194 (84.3)	36 (15.7)		
Internet				
No	19 (79.2)	5 (20.8)	‐	.789^a^
Yes	331 (81.3)	76 (18.7)		
Phone				
No	279 (79.5)	72 (20.5)	3.663	.056
Yes	71 (88.8)	9 (11.3)		
Text message				
No	228 (78.1)	64 (21.9)	5.791	.016
Yes	122 (87.8)	17 (12.2)		
Newspapers and magazines				
No	292 (79.8)	74 (20.2)	3.229	.072
Yes	58 (89.2)	7 (10.8)		
Community News				
No	238 (77.8)	68 (22.2)	8.127	.004
Yes	112 (89.6)	13 (10.4)		
Hospital training				
No	85 (72.6)	32 (27.4)	7.705	.006
Yes	265 (84.4)	49 (15.6)		
Position in this epidemic				
The quarantine ward	34 (75.6)	11 (24.4)	10.75	.041^a^
Triage station	6 (85.7)	1 (14.3)		
In preparation to contact high‐risk patients or work at the triage station	27 (81.8)	6 (18.2)		
Daily work in original department	268 (83.5)	53 (16.5)		
Fever clinic	10 (52.6)	9 (47.4)		
Administrative logistics personnel of the quarantine ward	5 (83.3)	1 (16.7)		
Wish most to receive support of “professional knowledge training” from the hospital				
No	96 (75)	32 (25)	4.596	.032
Yes	254 (83.8)	49 (16.2)		
Yes	170 (80.6)	41 (19.4)		
Wish most to receive support of “psychological counseling” from the hospital				
No	252 (84.6)	46 (15.4)	7.132	.008
Yes	98 (73.7)	35 (26.3)		
Yes	269 (82)	59 (18)		
Concerned about the preventive measures for the epidemic				
No	42 (68.9)	19 (31.1)	7.106	.008
Yes	308 (83.2)	62 (16.8)		
Yes	205 (82)	45 (18)		
Yes	330 (81.1)	77 (18.9)		
Worry most about the shortage of protective materials				
No	63 (72.4)	24 (27.6)	5.522	.019
Yes	287 (83.4)	57 (16.6)		
Yes	306 (81.8)	68 (18.2)		
The epidemic has changed your personal or family lifestyle				
No	101 (91)	10 (9)	9.378	.002
Yes	249 (77.8)	71 (22.2)		
The epidemic has played a positive role in improving the doctor–patient relationship				
No	275 (79.3)	72 (20.7)	4.462	.035
Yes	75 (89.3)	9 (10.7)		
Role change				
No	271 (84.2)	51 (15.8)	7.285	.007
Yes	79 (72.5)	30 (27.5)		

^a^ The Fisher exact test was used because the chi‐square test is invalid when the theoretical frequency of the cell is less than 5.

### 3.3 Factors affecting GHQ‐12 scores

**FIGURE 3:**
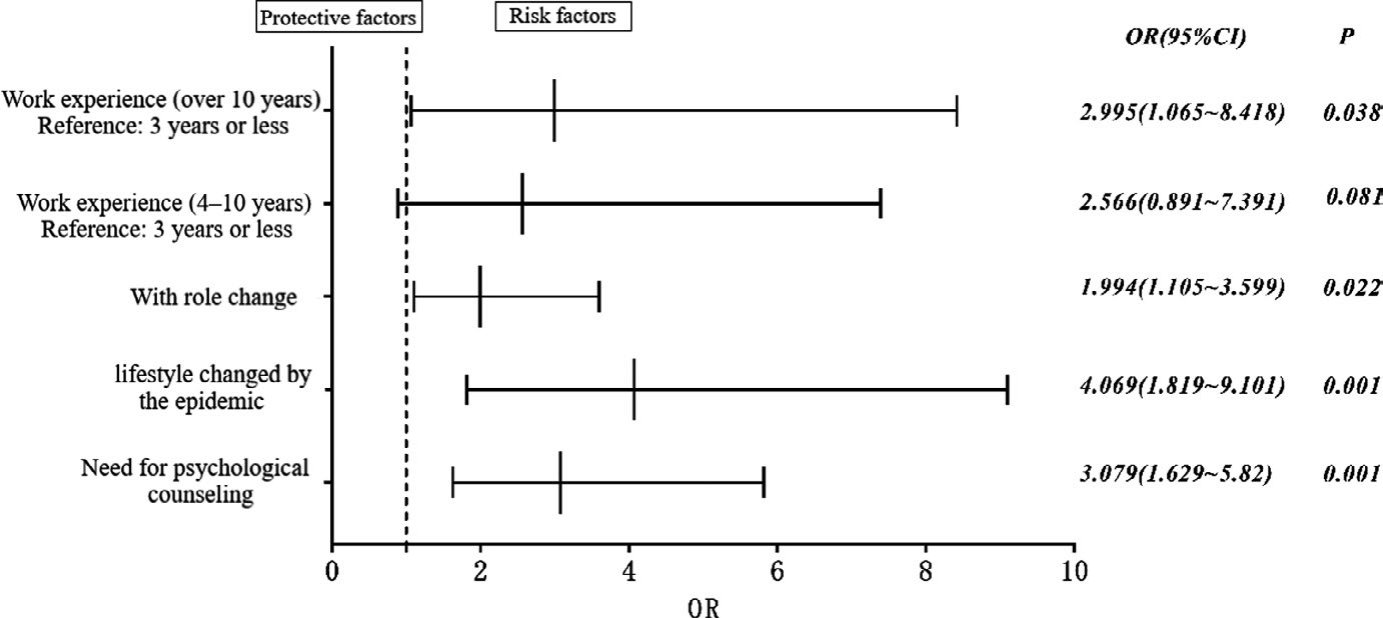
Multi‐factor binary logistic regression analysis. To assess the factors that affected the GHQ‐12 scores, we performed a binary logistic regression analysis to identify four factors that were significantly associated with the presence of emotional distress

To assess the factors that affected the GHQ‐12 scores, we performed a binary logistic regression analysis to identify factors that were significantly associated with the presence of emotional distress. This analysis resulted in four significant factors: (a) “work experience of 10 years or more” (OR 2.995; 95% CI: 1.065 to 8.418); (b) “the person's job position had changed” (OR 1.994; 95% CI: 1.105 to 3.599); (c) “lifestyle changed by the COVID‐19 outbreak” (OR 4.069; 95% CI: 1.819 to 9.101); (d) “need for psychological counseling” (OR 3.079; 95% CI: 1.629 to 5.82; Figure 3).

## 4 DISCUSSION

Our current study revealed an increased risk of developing psychological stress among healthcare workers with 10 years or more work experience. Nevertheless, there was no statistically significant difference among age groups at this experience level. Moreover, our analysis identified four factors that were significantly associated with elevated distress: (a) longer work experience, (b) change in job position, (c) change in lifestyle, and (d) need for psychological counseling. Thus, the data from our current study demonstrated that the COVID‐19 pandemic increased psychological stress among healthcare workers with longer work experience and job position changes. The weakness of our current study is the small sample size employed and the lack of long‐term follow‐up data. Future studies will investigate whether and how psychological counseling can help these healthcare workers to reduce their stress level and improve the quality of patient care.

Indeed, there was also an increase in severe psychological stress in healthcare workers with Middle East respiratory syndrome coronavirus (MERS)‐related positions during the 2015 outbreak (Lee et al., 2018). Our current study showed that the stress level was specifically higher in healthcare workers who had 10 years or longer work experience. The reason for this may be because this group of healthcare workers was required to make more decisions regarding diagnosis and treatment of COVID‐19 patients and therefore possessed more responsibilities and pressure. Such a difference may also be due to the setting of the healthcare work in China, for example, younger healthcare workers have fewer decisions to make during routine patient care than those in positions that are more senior. Moreover, our current study also revealed that job changes led to higher GHQ scores (≥ 3), which may be due to the fact that the sudden COVID‐19 outbreak and pandemic altered job position distribution. Nevertheless, the increase in distress level may not only have been directly involved with positions with potential high‐risk exposures, such as the diagnosis, treatment, and prevention of COVID‐19, but may have also involved job content after switching to new job functions. This conclusion is similar to the conclusions of Maunder et al. (2004) that found that some hospital workers who were assigned unfamiliar tasks appeared to be under a greater stress level than others who performed work with higher objective risk (i.e. nurses working in a severe acute respiratory syndrome isolation ward) even when the work was within their usual areas of competence and expertise.

The sudden COVID‐19 outbreak and pandemic greatly altered our lifestyle. Communities across the work restricted shop opening hours, individual movement, and gatherings, and often require wearing a facemask outside and maintaining elevated levels of personal hygiene. Healthcare workers, in addition to complying with these guidelines, need to also take further protective measurements and wear additional protective equipment during work. Moreover, they need to be separated from their family members and friends during such a difficult period. These lifestyle changes significantly impacted their routine, specifically in regard to job responsibility and social isolation, and notably affected their psychological health and stress level. Thus, prompt and continuous mental health intervention is needed to alleviate their stress and pressure. For example, a previous study showed that the severe acute respiratory syndrome (SARS) virus remained a fear among hospital workers 3 years after the epidemic (Wu et al., 2009). Our current study revealed that healthcare workers with the need for psychological counseling also faced more than a threefold increase in risk to develop psychological stress compared with those who did not need such psychological counseling.

However, the current study is preliminary and descriptive and much more examination needs to be performed. For example, regulations must be established on how to promptly identify those who need immediate psychological counseling and intervention or job changes. Our current study did not address how to improve and reduce the stress levels for these healthcare workers or provide any timely feedback on their needs. Moreover, our study was conducted in the early stage of the COVID‐19 pandemic in Xi'an Central hospital. With progression of the COVID‐19 pandemic and the subsequent formulation and implementation of various policies and measurements, the opinions and experience for healthcare workers may also change. Thus, further follow‐up studies using qualitative and quantitative methods are necessary. In addition, our current study employed an electronic questionnaire to collect data instead of a face‐to‐face questionnaire, resulting in some uncontrolled situations for the respondents to fill out the questionnaire in a self‐administered way, which may create a certain degree of subjectivity.

## 5 CONCLUSION

The results from the current study demonstrated that the COVID‐19 pandemic significantly impacted the psychological stress of healthcare workers. For example, those healthcare workers with more work experience (>10 years) experienced more risk in developing psychological stress than those with less work experience. Thus, speedy and continuous mental health interventions are needed to alleviate such stress and pressure to improve their mental health and patient care.

## CONFLICTS OF INTEREST

All authors declare that they have no conflicts of interests.

## AUTHOR CONTRIBUTION

Yongqin Li and Qiaoxia Wang participated in the design of this study. Qiaoxia Wang and Nan Wang carried out data analysis and statistical analysis. Caihong Lei and Yongqin Li carried out the study and collected important background information. Qiaoxia Wang drafted the manuscript. Nan Wang, Yuanyuan Liu, and Shanshan Zhu carried out literature search, data acquisition, and manuscript editing. Nan Wang performed manuscript review and revision. All authors have read and approved the content of the manuscript.

### Peer Review

The peer review history for this article is available at https://publons.com/publon/10.1002/brb3.2028.

## Data Availability

The data that support the findings of this study are available from the corresponding author upon reasonable request.
